# Supplementary material for: Theaflavins, polyphenols of black tea, inhibit entry of hepatitis C virus in cell culture
Source: PLoS One. 2018 Nov 28;13(11):e0198226. doi: 10.1371/journal.pone.0198226 (PMC6261387; doi:10.1371/journal.pone.0198226)
Supplement: S1 Fig — (A) Theaflavin (TF1) (a) chromatogram at 375 nm, Rt = 3.82 min, (b) UV spectrum, (c) mass spectrum in negative mode [M-H]- m/z 563; (B) Theaflavin-3-gallate (TF2) (a) chromatogram at 375 nm, Rt = 3.92 min, (b) UV spectrum, (c) mass spectrum in negative mode [M-H]- m/z 715; (C) Theaflavin-3,3'-digallate (a) chromatogram at 375 nm, Rt = 3.96 min, (b) UV spectrum, (c) mass spectrum in negative mode [M-H]- m/z 867. (PDF) [file pone.0198226.s001.pdf]

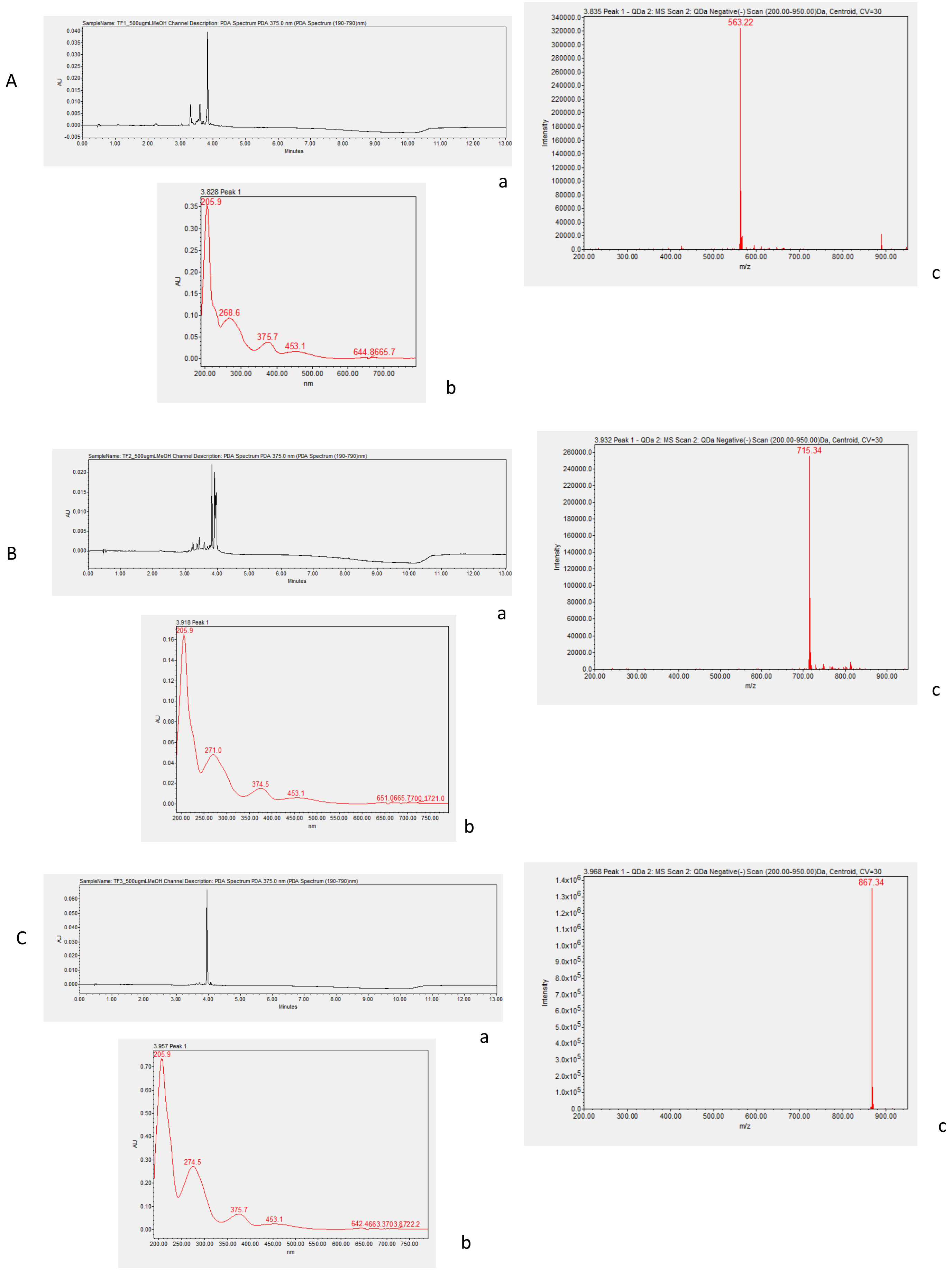

Figure S1. (A) Theaflavin (TF1) (a) chromatogram at 375 nm, Rt = 3.82 min , (b) UV spectrum, (c) mass spectrum in negative mode $[M-H]^-$ *m/z* 563; (B) Theaflavin-3-gallate (TF2) (a) chromatogram at 375 nm, Rt = 3.92 min, (b) UV spectrum, (c) mass spectrum in negative mode $[M-H]^-$ *m/z* 715 ; (C) Theaflavin-3,3'-digallate a) chromatogram at 375 nm, Rt = 3.96 min, (b) UV spectrum, (c) mass spectrum in negative mode $[M-H]^-$ *m/z* 867
